# Gut Microbiota and Related Metabolites Were Disturbed in Ulcerative Colitis and Partly Restored After Mesalamine Treatment

**DOI:** 10.3389/fphar.2020.620724

**Published:** 2021-01-18

**Authors:** Liang Dai, Yingjue Tang, Wenjun Zhou, Yanqi Dang, Qiaoli Sun, Zhipeng Tang, Mingzhe Zhu, Guang Ji

**Affiliations:** ^1^ Institute of Digestive Diseases, China-Canada Center of Research for Digestive Diseases (ccCRDD), Longhua Hospital, Shanghai University of Traditional Chinese Medicine, Shanghai, China; ^2^ School of Public Health, Shanghai University of Traditional Chinese Medicine, Shanghai, China

**Keywords:** 16S rRNA sequencing, metabolomics, mesalamine, ulcerative colitis, gut microbiota

## Abstract

Mesalamine has been well used in the improvement of ulcerative colitis (UC) in clinics, however, the underlying mechanisms were not well illustrated. To explore its efficacy from the perspective of gut microbiota and related metabolites, we employed 16S rRNA sequencing and metabolomics approaches in stool samples across 14 normal healthy controls (NC group), 10 treatment-naïve UC patients (UC group) and 14 UC patients responded to mesalamine treatment (mesalamine group). We noted that the gut microbiota diversity and community composition were remarkably perturbed in UC group and partially restored by mesalamine treatment. The relative abundance of 192 taxa in genus level were significantly changed in UC group, and 168 genera were significantly altered after mesalamine intervention. Meanwhile, a total of 127 metabolites were significantly changed in UC group and 129 metabolites were significantly altered after mesalamine treatment. Importantly, we observed that many candidates including 49 genera (such as *Escherichia-shigella, Enterococcus* and *Butyricicoccus*) and 102 metatoblites (such as isoleucine, cholic acid and deoxycholic acid) were reversed by mesalamine. Spearman correlation analysis revealed that most of the candidates were significantly correlated with Mayo score of UC, and the relative abundance of specific genera were significant correlated with the perturbation of metabolites. Pathway analysis demonstrated that genera and metabolites candidates were enriched in many similar molecular pathways such as amino acid metabolism and secondary metabolites biosynthesis. Importantly, ROC curve analysis identified a gut microbiota signature composed of five genera including *Escherichia-Shigella, Streptococcus, Megamonas, Prevotella_9* and [*Eubacterium*] *_coprostanoligenes _group* which might be used to distinguish UC group from both NC and mesalamine group. In all, our results suggested that mesalamine might exert a beneficial role in UC by modulating gut microbiota signature with correlated metabolites in different pathways, which may provide a basis for developing novel candidate biomarkers and therapeutic targets of UC.

## Introduction

Ulcerative colitis (UC) is a chronic inflammatory bowel disease characterized by relapsing and remitting mucosal inflammation. The affected site starts in the rectum and could extend to proximal segments of the colon (Ungaro et al., 2017). The typical symptoms of UC include bloody stools, diarrhoea and fatigue, which may severely impact work capacity and quality of life (Hoivik et al., 2013; Lynch and Hsu, 2020). The global incidence and prevalence of UC experienced a great increase in recent years, posing a significant burden on public health system (Kotze et al., 2020). Hence, it is urgent to explore underlying pathogenesis of UC and discover efficient therapies.

UC is consider as a disease of unknown aetiology, which is a multifactorial disorder (Shen et al., 2018; Kaur and Goggolidou, 2020). It has been reported that a variety of complex factors including genetics, environment, epithelial barrier defects and immune system disorders were related to the pathogenesis of ulcerative colitis (Porter et al., 2020). Meanwhile, abundant evidence have demonstrated that gut microbiota might play a crucial role in ulcerative colitis, and many studies have revealed that biodiversity and composition of gut microbiota were changed in UC patients and animal models (Bajer et al., 2017; Shang et al., 2017). However, the mechanisms of gut microbiota contributing to the pathogenesis of UC and efficient interventions need further investigation. The aim of UC treatment is to induce and maintain remission of the disease (Scaldaferri et al., 2016). Mesalamine has been used in controlling UC, which is recommended as the first-line therapy (Adams and Bornemann, 2013). As a free radical scavenger and an antioxidant, mesalamine could regulate inflammatory response by modulating the production of inflammatory cytokines and the functions of immune cells (Nakashima and Preuss, 2020). However, few studies have reported the influence of mesalamine on gut microbiota and related metabolites in UC patients.

In the present study, we employed 16S rRNA sequencing and LC-MS (liquid chromatography mass spectrometry) metabolomics, observed the changes of gut microbiota composition and the related metabolites among normal healthy controls, UC patients with no treatment and UC patients with mesalamine treatment, reported the effects of mesalamine in restoring perturbance of metabolites and gut microbiota, and identified the underlying functional pathways and biomarkers of UC. The present study will enhance the comprehension of gut microbiota in UC pathogenesis and mechanisms of mesalamine in treating UC, which may benefit development of novel therapeutic agents in future.

## Materials and Methods

### Study Design

This exploratory study composed of two sequential cross-sectional trials. Firstly, we designed a cohort contained 10 treatment-naïve UC patients (UC group) and 14 healthy volunteers (NC group), to investigate the potential difference in gut microbiota and related metabolites between UC and healthy status. Then, another cohort was established, which composed of all patients from UC group and 14 UC patients in mesalamine group who were well responded to mesalamine treatment, to discover underlying gut microbiota and related metabolites that mesalamine could modulate. All patients were screening from inpatient and outpatient of gastroenterology and anorectal departments in Longhua Hospital from January 2019 to August 2020. Healthy controls were recruited voluntarily from health examination department. The present study was approved by the Ethics Committee of Longhua Hospital, and informed consent was obtained from all participants.

### Participants and Sample Collection

UC was diagnosed based on a combination of clinical symptoms, endoscopic and histological findings, and absence of other reasons induced colitis according to the Chinese consensus on diagnosis and treatment of inflammatory bowel disease (Beijing, 2018) (Wu et al., 2018). In brief, the following criteria were compulsory: 1) persistent or recurrent diarrhea with mucus and bloody purulent discharge for more than 6 weeks; 2) endoscopic findings of erythema, mucosal congestion, disappearance of vascular pattern, erosions, ulcerations and so on; 3) histological evidences of inflammatory cell infiltration, distortion of crypt architecture and mucosal erosion or ulceration; 4) exclusion of other pathologies including but not limited to infection, medications, radiation and ischemia.

Patients aged 18–65 years old who had mild and moderate UC with a modified Mayo score of 3–10 were screened. The additional inclusion criteria for UC group were treatment-naïve patients, who were defined as patients who were initially diagnosed as UC and received no treatment, or had a complete remission at least 6 months but experienced a recent relapse before any medication administration. On the other hand, the extra inclusion criteria for mesalamine group were mesalamine-responded UC patients. The administration rules should be oral intake of 1.0 g mesalazine enteric-coated tablets, three times a day, 1 h before three meals for at least continuous 3 months. The treatment response was set as a reduction from initial treatment in total Mayo score of at least 30%, with an accompanying decline in the dimension for rectal bleeding of at least one point or an absolute score for rectal bleeding of 0 or 1 (Rutgeerts et al., 2005). Patients were excluded if they met any of following criteria: breastfeeding or pregnant, participation in other clinical trials within the past 6 months, administration of antibiotics within the past 3 months, administration of immunosuppressive agents, biological agents, other non-steroidal (steroidal) anti-inflammatory drugs besides mesalamine, gastrointestinal mucosal protective agents, intestinal probiotics and prebiotics within the past 4 weeks. Besides, patients were also excluded if they combined with severe heart, liver, kidney and other important organ and blood system diseases, as well as gastroduodenal ulcer, history of intestinal surgery, intestinal obstruction, intestinal perforation, perianal abscess, severe hemorrhagic disease and mental disorders. This study also recruited age and gender matched healthy volunteers as NC group. The corresponding eligible criteria contained: 1) no administration of salicylic acid drugs in the past 4 weeks; 2) no history of gastrointestinal diseases in the past 6 months; 3) no existence of gastrointestinal symptoms such as abdominal distension, abdominal pain, diarrhea and constipation; 4) no combination with severe cardiovascular, hepatic, renal and digestive diseases. Based on above inclusion and exclusion criteria, the first cohort included 10 treatment-naïve UC patients and 14 healthy volunteers, then the second cohort contained 10 treatment-naïve UC patients and 14 mesalamine-responded UC patients.

Age, gender and body mass index (BMI) were recorded for all participants. Disease course, Montreal classification, Mayo score and corresponding disease severity were also documented for UC patients. In addition, Baron index was utilized to quantify the endoscopic activity in UC patients (Baron et al., 1964). Participants were informed to terminate administration of antibiotics, probiotics, prebiotics or other microbiota-related preparations at least 4 weeks before sampling. All participants were instructed to collect 3.0 g morning first feces using sterile fecal collection tubes. The fecal samples should avoid contamination from other mediums such as urine. The collected samples were stored at −80°C for further16S rRNA sequencing and untargeted metabonomics detection.

### DNA Extraction and 16S rRNA Sequencing

Fecal samples were performed 16S rRNA sequencing in Shanghai Meiji Biomedical Technology Co., Ltd. (Shanghai, China) following the manufactures’ procedures. Briefly, DNA were extracted from fecal samples according to the instructions of E. Z.N.A^®^soil (Omega Bio-tek, Norcross, GA, United States). The quality of DNA was detected by 1% agarose gel electrophoresis, and the concentration and purity were detected by NanoDrop 2000. 16S rRNA genes were amplified using PCR with primers targeting the V3-V4 region (338F–806R). The amplicons were purified using AxyPrep DNA Gel Extraction Kit (Axygen Biosciences, Union City, CA, United States), and quantified by QuantiFluor™-ST (Promega, United States) to construct libraries and perform paired-end rRNA sequencing on Illumina MiSeq platform (Illumina, San Diego, United States) following the manufactures’ instructions.

### 16S rRNA Sequencing Data Analysis

The adapters and low-quality bases were trimmed using fastp tool to obtain clean reads (Chen et al., 2018), and then merged by FLASH software (Magoc and Salzberg, 2011). Sequences with 97% similarity were clustered in OTUs (operational taxonomic units) using UPARSE software (Edgar, 2013). Taxonomy was annotated and aligned with Silva 16S-rDNA database (v138) using RDP classifier (Wang et al., 2007). The Chao and Shannon index was used to estimate Alpha diversity and principle coordinates analysis (PCoA) using unweighted UniFrac distance was performed to reveal the Beta diversity among samples. ANOSIM (analysis of similarities) analysis was performed to assess the significance of difference in PCoA plots. Kruskal-Wallis H test or Wilcoxon rank-sum test were used to assess the significant difference of bacterial genera abundance among groups. The functional prediction of taxa was performed using PICRUSt analysis (Langille et al., 2013). *p* value less than 0.05 was considered as significantly different. The signature to discriminate UC group from NC and mesalamine group was identified using ROC (receiving operator curve) analysis by estimating AUC (area under curve) value.

### Metabolomics Data Acquisition

Stool samples were performed metabolomics analysis in Shanghai Meiji Biomedical Technology Co., Ltd. (Shanghai, China) following the manufactures’ procedures. Briefly, 50 mg stool samples were added 400 Ul of cold methanol solution (methanol: water = 4:1), broken by a high-throughput tissue crusher at low temperature. After vortex mixing, the samples were extracted by ultrasound on ice for 10 min and three times, placed at −20°C for 30 min, centrifuged at 13,000 g, 4°C for 15 min, and the supernatant was performed metabolomics analysis using ultra performance liquid chromatography-triple time of flight mass spectrometry (UPLC-Triple TOF-MS, AB SCIEX Company, United States) following the manufactures’ protocols. The chromatographic column was BEHC18 column (100 × 2.1 mm i.d., 1.7 µm; Waters, Milford, United States). Mobile phase A was water (containing 0.1% formic acid), and mobile phase B was acetonitrile/isopropanol (1/1) (containing 0.1% formic acid). The flow rate was 0.4 ml/min, injection volume was 20 µl and column temperature was 40°C. The chromatographic elution gradient was 0.0 min, 95%A and 5% B; 3 min, 80%A and 20% B; 9.0 min, 5%A and 95% B; 13 min, 5%A and 95% B; 13.1 min, 95%A and 5% B and 16 min, 95%A and 5% B. The samples mass spectrometry signal acquisition was used positive and negative ion scanning mode. The electrospray capillary voltage, injection voltage and collision voltage were 1.0 kV, 40 V and 6 eV, respectively. The ion source temperature and desolvation temperature were 120 °C and 500 °C, carrier gas flow was 900 L/h, mass spectrometry scanning range was 50–1,000 m/z and resolution was 30,000. Quality control (QC) samples were pooled from all experimental samples and analyzed with the same procedure.

### Metabolomics Data Analysis

Raw data were processed using Progenesis QI software (Waters Corporation, Milford, United States), then a data matrix of retention time, m/z and peak area was obtained. Only variables with a non-zero value of more than 50% in all samples were retained, and the missing values were filled with 1/2 of the minimum value in the original matrix. The total peaks were normalized, and variables with a relative standard deviation (RSD) of QC samples of more than 30% were deleted to obtain a data matrix for further analysis. To identify the structure of metabolites, raw data were imported to Progenesis QI software (Waters Corporation, Milford, United States) and the mass spectra were compared to an in-house standard library and public databases such as The Human Metabolome Database (HMDB) and METLIN.

Using R package ropls (version1.6.2), principle component analysis (PCA) and orthogonal partial least squares discriminant analysis (OPLS-DA) were performed for multivariate statistical analysis, and variable importance of projection (VIP) values were obtained. For univariate statistical analysis, Welch’s *t* test was used to calculate *p* values between groups. Fold changes of metabolites were calculated between groups based on the ratio of average normalized peak intensity. The differential metabolites between groups were identified with the threshold of VIP > 1 and *p* < 0.05. Venn diagram (R package, version1.6.2) was used to obtain the intersection of differential metabolites between pairwise groups. Hierarchical cluster was performed to reveal the expression patterns of differential metabolites among groups, and pathway enrichment was performed to reveal metabolites related biological functions using scipy (Python, version1.0.0).

### Statistical Analysis

For baseline data, data were presented as median with interquartile range or number with percentage. Statistical analysis was performed using SPSS 24.0 software. The qualitative data among three groups were assessed using one-way analysis of variance (ANOVA) or Kruskal-Wallis test based on data distribution. The qualitative data was analyzed by Chi-square test. Difference between two groups were determined by Student’s T test or Wilcoxon rank sum test. For correlation analysis, Spearman rank correlation coefficient was calculated. *p* value less than 0.05 was considered as significant difference.

## Results

### Baseline Characteristic of Participants

A total of 14 normal healthy controls (NC group), 10 UC patients with no treatment (UC group), and 14 UC patients with mesalamine treatment (mesalamine group) were included in the present study. Detailed demographic and clinical characteristics of enrolled participants were shown in Table 1. No significant difference was found in age, gender and BMI among three groups. UC phenotypes based on Montreal Classification was comparable between UC group and mesalamine group. Patients in mesalamine group showed milder disease severity and endoscopic activity than UC groups, according to modified Mayo score and Baron index, respectively.

**TABLE 1:** Baseline characteristics of included participants.

	NC group (n = 14)	UC group (n = 10)	Mesalamine group (n = 14)	*p* Value
Age, years	32.50 (10.0)	27.50 (11.0)	42.50 (23)	0.084
Gender, male	7 (50.0%)	5 (50.0%)	9 (64.3%)	0.694
BMI, kg/m^2^	22.35 (1.5)	20.15 (4.4)	23.35 (2.5)	0.075
Course, months	—	9.50 (34.0)	48.00 (77.0)	0.007
Montreal classification				0.546
Proctitis	—	3	6	
Left-sided colitis	—	3	4	
Extensive colitis	—	4	4	
Modified mayo score	—	8.00 (3.0)	5.00 (2.0)	0.005
Disease severity^a^	—			0.022
Clinical remission	—	0	2	
Mild	—	3	9	
Moderate	—	6	3	
Severe	—	1	0	
Baron index	—	3.00 (0.0)	1.00 (2.0)	0.002

^a^ Evaluation of disease severity is based on modified Mayo score: 1) Clinical remission: total score ≤2 points, and every sub-dimension score ≤1 point; 2) Mild: total score of three to five points; 3) Moderate: total score of 6–10 points; 4) Severe: total score of 11–12 points.

### Mesalamine Intervention Improved Gut Microbiota Diversity in UC Patients

**FIGURE 1:**
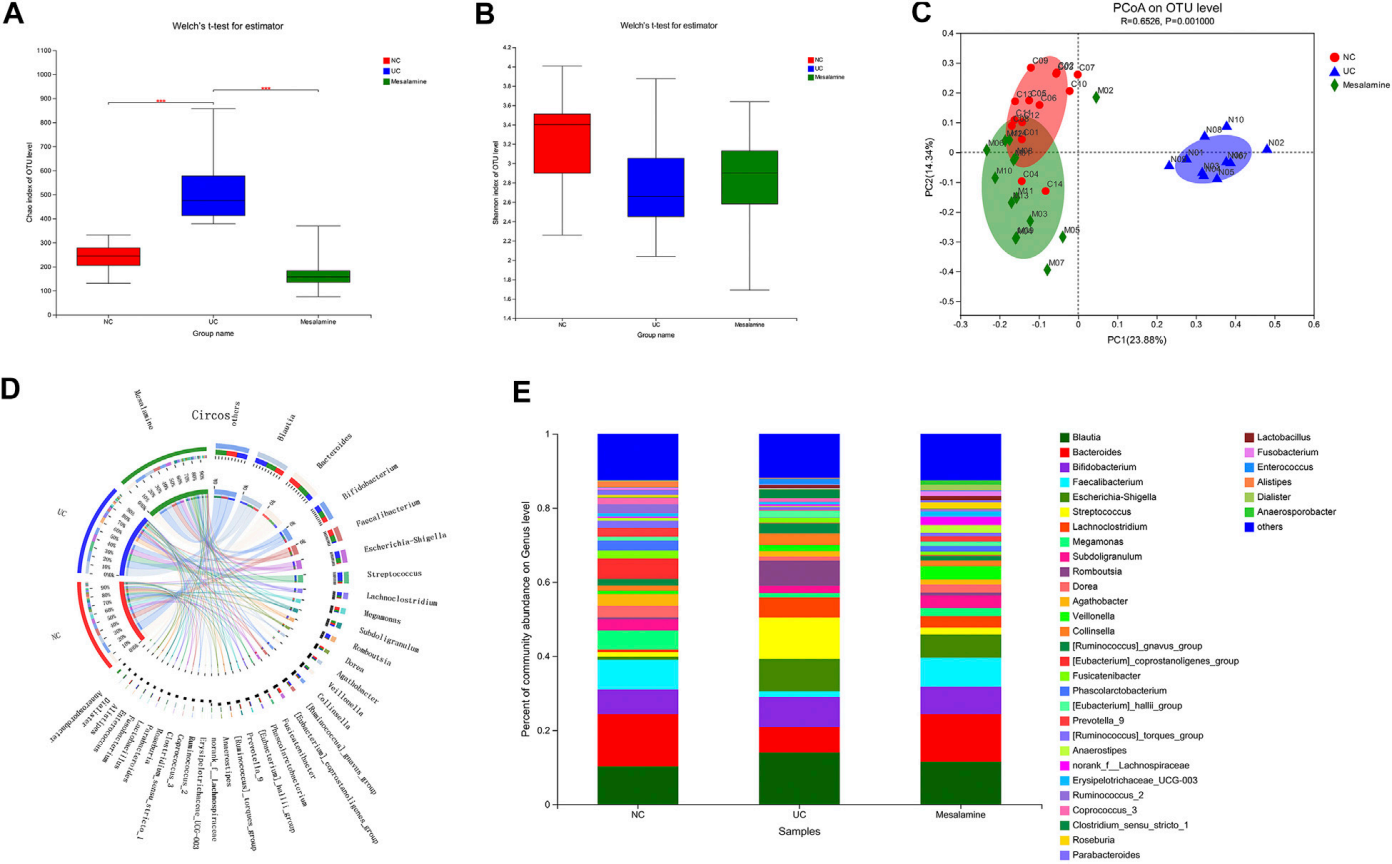
Gut microbiota diversity and composition analysis. **(A)** α-Diversity evaluated by Chao index (***p < 0.001) **(B)**. α-Diversity evaluated by Shannon index. **(C)** PCoA plots based on unweighted UniFrac distance **(D)**. Diagram of Circos analysis, the small semicircle **(left half circle)** represents the species composition in the sample, the color of outer ribbon represents the group from which the species came, the color of inner ribbon represents the species, and the length represents the relative abundance of the species in the corresponding samples; the large semicircle **(right half circle)** represents the distribution proportion of species in different samples at the taxonomic level, and the outer layer ribbon represents the species, the inner ribbon color represents different groups, and the length represents the distribution proportion of the sample in a certain species. **(E)** The overall percentage of gut microbiota community abundance on genus level.

The 16S rRNA sequencing of stool samples from three groups was performed to reveal the difference of gut microbial community structure. Analysis of Chao index indicated that there was a significant increase in taxa richness in UC group, while mesalamine could obviously restore the disturbance (Figure 1A). The Shannon index of taxa evenness exhibited a decreased trend in UC group, and mesalamine partially improved the perturbance (Figure 1B). PCoA plots revealed that there was remarkable difference in bacterial composition between UC group and NC group, while mesalamine intervention could ameliorate the difference (Figure 1C).

### Mesalamine Intervention Improved Gut Microbiota Abundance in UC Patients

**FIGURE 2:**
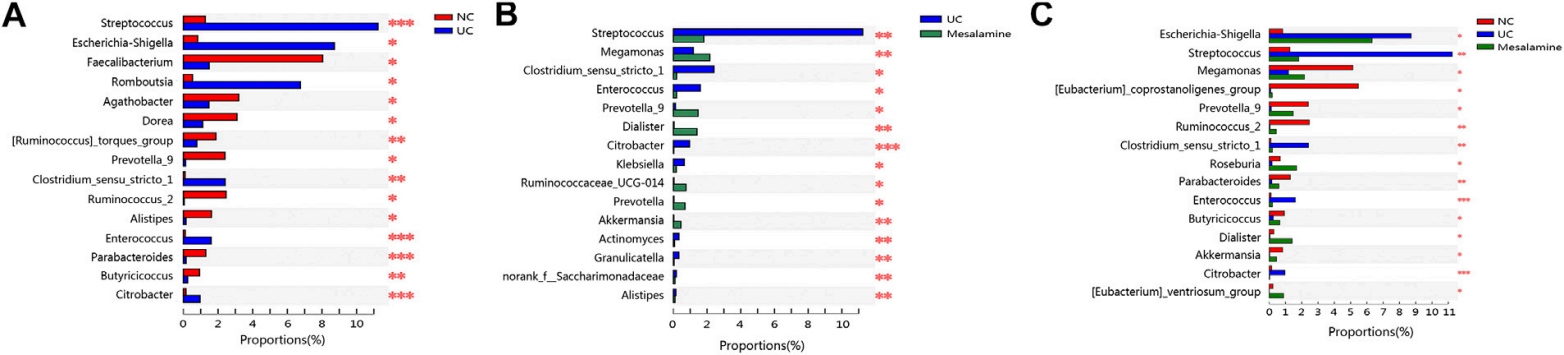
Significantly changed taxa on genus level among groups. **(A)** Top 15 differential genera between UC group and NC group. **(B)** Top 15 differential genera between UC group and mesalamine group **(C)**. Top 15 mesalamine restored genera. *0.01 < *p* ≤ 0.05, **0.001 < *p* ≤ 0.01, ***p ≤ 0.001.

To examine whether relative abundance of gut microbiota was associated with the diversity difference, Circos analysis (Figure 1D) and community bar plots (Figure 1E) at genus level were performed. The results showed that the relative abundance of various specific microbiotas at genus level were different between groups. For example, the relative abundance of genus *Streptococcus* in UC group, mesalamine group and NC group was 11.24, 1.81, and 1.27%, respectively. To test whether there was a significant difference of specific microbiota between UC group and NC group or between mesalamine group and UC group, the differential microbiota analysis based on relative abundance at genus level was performed using Wilcoxon rank-sum test. Our results showed that there were 192 significantly differential genera between UC group and NC group (Supplementary Table S1), and the representative top 15 genera were presented in Figure 2A. Meanwhile, mesalamine intervention significantly changed the relative abundance of 168 genera in UC patients (Supplementary Table S2), and the top 15 differential genera were revealed in Figure 2B. Interestingly, we observed that mesalamine intervention could significantly reverse the relative abundance of 49 genera in UC patients (Table 2), which were identified as candidate genera for further analysis. The top 15 mesalamine reversed genera were presented in Figure 2C, which revealed that the relative abundance of *Escherichia-Shigella, Megamonas, Clostridium_ sensu_stricto_1, Enterococcus* and *Citrobacter* was significantly increased in UC group compared to NC, and restored by mesalamine treatment. The relative abundance of genera including *Megamonas* [*Eubacterium*]_*ventriosum_ group, Prevotella_9, Ruminococcus_2, Roseburia, Parabacteroides, Butyricicoccus, Dialister, Akkermansia* and [*Eubacterium*]*_coprostanoligenes_group* were significantly reduced in UC group compared to NC, which was obviously increased by mesalamine intervention.

**TABLE 2:** 49 candidate genera reversed by mesalamine intervention.

Genus name	NC group proportion (%)	UC group proportion (%)	Mesalamine group proportion (%)	*p* Value
g__Escherichia-shigella	0.827	8.721	6.317	0.039
g__Megamonas	5.136	1.185	2.162	0.017
g__Streptococcus	1.266	11.240	1.807	0.001
g__Roseburia	0.669	0.171	1.676	0.042
g__Prevotella_9	2.401	0.128	1.470	0.020
g__Dialister	0.281	0.044	1.406	0.024
g__[Eubacterium]_ventriosum_group	0.229	0.003	0.877	0.020
g__Butyricicoccus	0.931	0.247	0.648	0.017
g__Parabacteroides	1.299	0.158	0.595	0.003
g__Akkermansia	0.824	0.032	0.449	0.010
g__Ruminococcus_2	2.465	0.050	0.431	0.002
g__Clostridium_sensu_stricto_1	0.102	2.413	0.194	0.008
g__Enterococcus	0.109	1.602	0.193	0.001
g__[Eubacterium]_coprostanoligenes_group	5.477	0.092	0.186	0.016
g__Klebsiella	0.228	0.648	0.176	0.026
g__[Clostridium]_innocuum_group	0.007	0.533	0.153	0.008
g__Ruminococcaceae_UCG-002	0.418	0.009	0.153	0.046
g__norank_f__Saccharimonadaceae	0.009	0.180	0.102	0.000
g__*Actinomyces*	0.048	0.335	0.070	0.002
g__Odoribacter	0.152	0.014	0.058	0.046
g__Pseudomonas	0.609	0.012	0.055	0.006
g__Coprococcus_1	0.109	0.029	0.053	0.023
g__Ruminococcaceae_UCG-003	0.085	0.005	0.049	0.045
g__Rothia	0.006	0.131	0.045	0.000
g__Granulicatella	0.015	0.331	0.044	0.001
g__Family_XIII_AD3011_group	0.038	0.008	0.044	0.003
g__*Citrobacter*	0.150	0.963	0.029	0.000
g__Bilophila	0.092	0.005	0.028	0.012
g__unclassified_f__Ruminococcaceae	0.038	0.061	0.020	0.009
g__Gemella	0.004	0.148	0.018	0.000
g__Oribacterium	0.002	0.023	0.014	0.025
g__norank_f__Muribaculaceae	0.080	0.176	0.013	0.000
g__Solobacterium	0.002	0.038	0.008	0.000
g__Morganella	0.000	0.011	0.006	0.004
g__Atopobium	0.003	0.021	0.005	0.026
g__Staphylococcus	0.001	0.149	0.003	0.000
g__Lachnospiraceae_UCG-001	0.144	0.002	0.003	0.008
g__Weissella	0.002	0.030	0.002	0.012
g__*Aeromonas*	0.000	0.049	0.001	0.000
g__Corynebacterium	0.002	0.022	0.001	0.028
g__Lactococcus	0.004	0.021	0.001	0.000
g__Family_XIII_UCG-001	0.031	0.000	0.001	0.003
g__Bacillus	0.000	0.181	0.001	0.000
g__Corynebacterium_1	0.001	0.017	0.001	0.004
g__Finegoldia	0.001	0.012	0.001	0.013
g__*Acinetobacter*	0.001	0.033	0.000	0.000
g__Porphyromonas	0.002	0.021	0.000	0.000
g__Leuconostoc	0.002	0.008	0.000	0.004
g__Anaerofilum	0.001	0.002	0.000	0.028

### Mesalamine Intervention Restored the Perturbance of Fecal Metabolites in UC Patients

**FIGURE 3:**
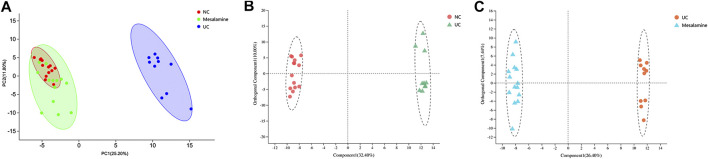
PCA and OPLS-DA plots among groups. **(A)** PCA score plots of NC, UC and mesalamine groups, red dots represented NC group, green dots represented mesalamine group and blue dots represented UC group. **(B)** OPLS-DA score plots of NC and UC groups, red dots represented NC group and green triangles represented UC group. **(C)** OPLS-DA score plots of UC and mesalamine groups, red dots represented UC group and blue triangles represented mesalamine group.

**FIGURE 4:**
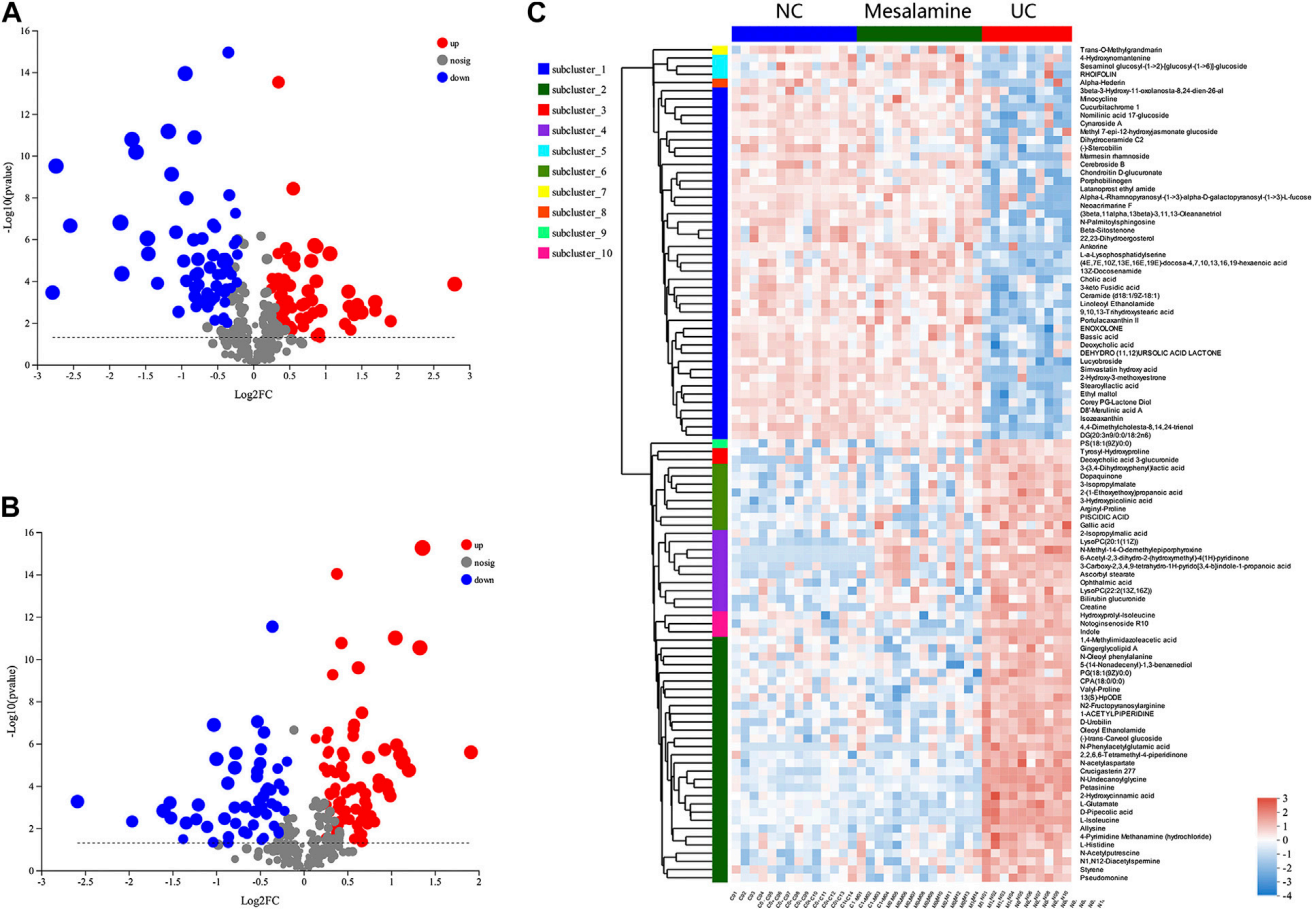
Significantly changed metabolites among groups. **(A)** Volcano plots of differential metabolites between UC group and NC group, grey dots represent metabolites with no significant change, red dots represent up-regulated metabolites and green dots represent down-regulated metabolites. **(B)** Volcano plots of differential metabolites between UC group and mesalamine group, grey dots represent metabolites with no significant change, red dots represent up-regulated metabolites and green dots represent down-regulated metabolites. **(C)** Hierarchical cluster of 102 candidate metabolites. The color indicates the relative expression level of metabolites in this group of samples, red indicates up-regulated and blue indicated down-regulated.

Using UPLC-Triple TOF-MS, the fecal metabolites across NC, UC and mesalamine groups were profiled. As shown in Figure 3A, the PCA plots of three groups revealed that there was distinct separation among groups, and the plots of mesalamine group were close to NC group. The results indicated that the metabolites profile in UC group might be different from both NC and mesalamine group, and the metabolites profile of latter two groups might be similar. To identify the differential metabolites, OPLS-DA model was performed between pairwise groups (UC vs NC group, and mesalamine vs UC group). The results showed that the plots of UC group samples were obviously separated from NC or mesalamine group (Figures 3B,C). With the threshold of VIP more than 1, *p* value less than 0.05 and fold change not equal to 1, a total of 127 differential metabolites between UC group and NC were obtained, and 129 differential metabolites between mesalamine and UC group were identified (Figures 4A,B; Supplementary Table S3, S4). Importantly, a total of 102 metabolites reversed by mesalamine intervention in UC patients were filtered out as candidates for further analysis (Figure 4C; Table 3). For example, the level of ophthalmic acid, isoleucine, styrene and creatine was elevated in treatment-naïve UC patients compared to normal healthy controls, and restored by mesalamine treatment. The level of cholic acid, deoxycholic acid and enoxolone was reduced in treatment-naïve UC patients, and restored by mesalamine intervention.

**TABLE 3:** 102 candidate metabolites reversed by mesalamine intervention.

Metabolite	Fold change (UCgroup/NC group)	UCgroup vs NC group *p* value	Fold change (mesalamine group/UC group)	Mesalamine group vs UC group *p* Value
D8′-merulinic acid A	0.573	0.000	1.726	0.000
(4E,7E,10Z,13E,16E,19E)-docosa-4,7,10,13,16,19-hexaenoic acid	0.731	0.000	1.443	0.000
13(S)-HpODE	1.234	0.000	0.734	0.000
Marmesin rhamnoside	0.309	0.001	2.865	0.003
Cynaroside A	0.570	0.001	1.728	0.001
Methyl 7-epi-12-hydroxyjasmonate glucoside	0.530	0.032	1.819	0.048
2-(1-Ethoxyethoxy)propanoic acid	1.296	0.000	0.761	0.001
Valyl-Proline	1.243	0.000	0.638	0.000
Ophthalmic acid	1.750	0.000	0.587	0.003
Neoacrimarine F	0.400	0.000	2.305	0.001
N-acetylaspartate	1.342	0.000	0.727	0.000
N2-Fructopyranosylarginine	2.104	0.000	0.479	0.000
l-Histidine	1.275	0.000	0.740	0.000
Pseudomonine	1.693	0.000	0.458	0.000
Stearoyllactic acid	0.545	0.000	1.824	0.000
N-Palmitoylsphingosine	0.768	0.000	1.296	0.000
Cerebroside B	0.770	0.000	1.289	0.001
Lucyobroside	0.718	0.001	1.423	0.001
(-)-Stercobilin	0.624	0.001	1.373	0.029
Hydroxyprolyl-isoleucine	1.330	0.006	0.717	0.001
Gallic acid	1.286	0.010	0.774	0.016
3-Hydroxypicolinic acid	1.355	0.000	0.777	0.000
3-Isopropylmalate	1.704	0.000	0.607	0.001
3-(3,4-Dihydroxyphenyl)lactic acid	1.953	0.000	0.624	0.002
Piscidic acid	1.634	0.000	0.503	0.000
Dopaquinone	1.276	0.000	0.810	0.000
Portulacaxanthin II	0.544	0.000	2.037	0.000
2-Isopropylmalic acid	1.327	0.000	0.784	0.000
Rhoifolin	0.546	0.041	2.149	0.009
Cholic acid	0.801	0.007	1.221	0.014
2,2,6,6-Tetramethyl-4-piperidinone	1.204	0.000	0.836	0.000
Nomilinic acid 17-glucoside	0.522	0.003	1.872	0.004
Tyrosyl-Hydroxyproline	1.733	0.002	0.629	0.019
Simvastatin hydroxy acid	0.478	0.000	1.996	0.000
Notoginsenoside R10	1.745	0.000	0.518	0.000
Deoxycholic acid	0.780	0.004	1.218	0.017
DG (20:3n9/0:0/18:2n6)	0.555	0.000	1.711	0.000
Ethyl maltol	0.741	0.000	1.330	0.000
Sesaminol glucosyl-(1->2)-[glucosyl-(1->6)]-glucoside	0.267	0.008	3.894	0.005
Ceramide (d18:1/9Z-18:1)	0.759	0.000	1.349	0.000
N-Oleoyl phenylalanine	1.558	0.000	0.551	0.000
CPA(18:0/0:0)	1.703	0.000	0.464	0.000
LysoPC(20:1 (11Z))	2.195	0.000	0.763	0.001
Ascorbyl stearate	3.090	0.000	0.696	0.004
3beta-3-Hydroxy-11-oxolanosta-8,24-dien-26-aL	0.618	0.015	1.606	0.015
Dihydroceramide C2	0.593	0.006	1.566	0.018
Enoxolone	0.740	0.003	1.397	0.001
Ankorine	0.368	0.001	2.885	0.001
Oleoyl ethanolamide	1.902	0.000	0.598	0.000
2-Hydroxy-3-methoxyestrone	0.144	0.000	6.006	0.001
N-Phenylacetylglutamic acid	6.671	0.000	0.266	0.000
3-keto Fusidic acid	0.721	0.001	1.343	0.002
5-(14-Nonadecenyl)-1,3-benzenediol	1.305	0.000	0.683	0.000
Cucurbitachrome 1	0.397	0.002	2.343	0.004
Bilirubin glucuronide	2.746	0.000	0.635	0.005
13Z-Docosenamide	0.785	0.000	1.281	0.000
Gingerglycolipid A	1.314	0.001	0.552	0.000
Petasinine	2.264	0.000	0.484	0.000
l-isoleucine	1.926	0.000	0.390	0.000
9,10,13-Trihydroxystearic acid	0.675	0.000	1.448	0.000
(-)-trans-Carveol glucoside	1.410	0.000	0.724	0.000
Indole	1.516	0.000	0.398	0.000
*Trans*-O-Methylgrandmarin	0.309	0.003	2.599	0.033
N-Methyl-14-O-demethylepiporphyroxine	3.542	0.000	0.670	0.032
4-Hydroxynornantenine	0.737	0.008	1.477	0.002
Styrene	1.740	0.000	0.613	0.000
Alpha-l-Rhamnopyranosyl-(1->3)-alpha-d-galactopyranosyl-(1->3)-l-fucose	0.687	0.019	1.397	0.036
6-Acetyl-2,3-dihydro-2-(hydroxymethyl)-4(1H)-pyridinone	3.212	0.000	0.615	0.001
Arginyl-Proline	1.773	0.000	0.662	0.001
Porphobilinogen	0.560	0.003	1.714	0.006
Chondroitin d-glucuronate	0.609	0.001	1.490	0.007
1-Acetylpiperidine	1.341	0.000	0.715	0.000
N-Acetylputrescine	1.471	0.000	0.812	0.002
N1,N12-Diacetylspermine	1.426	0.000	0.740	0.001
1,4-Methylimidazoleacetic acid	1.298	0.000	0.746	0.001
Creatine	3.589	0.000	0.434	0.000
4-Pyrimidine methanamine (hydrochloride)	1.472	0.000	0.673	0.000
d-Pipecolic acid	1.260	0.000	0.741	0.000
Beta-sitostenone	0.841	0.000	1.169	0.000
Corey PG-Lactone Diol	0.590	0.000	1.586	0.001
L-a-Lysophosphatidylserine	0.799	0.000	1.403	0.000
PG (18:1 (9Z)/0:0)	1.171	0.000	0.813	0.000
Alpha-Hederin	0.413	0.011	2.049	0.046
Linoleoyl ethanolamide	0.677	0.000	1.445	0.000
N-Undecanoylglycine	1.271	0.000	0.768	0.000
Bassic acid	0.822	0.000	1.214	0.000
Minocycline	0.690	0.002	1.459	0.002
Latanoprost ethyl amide	0.353	0.003	3.037	0.001
22,23-Dihydroergosterol	0.787	0.000	1.229	0.000
3-Carboxy-2,3,4,9-tetrahydro-1H-pyrido [3,4-b]indole-1-propanoic acid	5.817	0.000	0.624	0.044
Dehydro (11,12)ursolic acid lactone	0.704	0.000	1.377	0.000
Crucigasterin 277	1.761	0.000	0.649	0.000
D-Urobilin	2.769	0.000	0.465	0.000
l-Glutamate	1.187	0.000	0.795	0.000
4,4-Dimethylcholesta-8,14,24-trienol	0.681	0.000	1.369	0.000
Allysine	1.442	0.000	0.675	0.000
PS(18:1 (9Z)/0:0)	1.673	0.001	0.723	0.004
(3beta,11alpha,13beta)-3,11,13-Oleananetriol	0.379	0.004	2.537	0.006
Deoxycholic acid 3-glucuronide	2.053	0.003	0.509	0.000
LysoPC(22:2 (13Z,16Z))	1.371	0.000	0.775	0.007
Isozeaxanthin	0.712	0.000	1.412	0.000
2-Hydroxycinnamic acid	1.165	0.000	0.824	0.000

### Mesalamine Restored Gut Microbiota and Metabolites Correlated with UC Clinical Indexes

**FIGURE 5:**
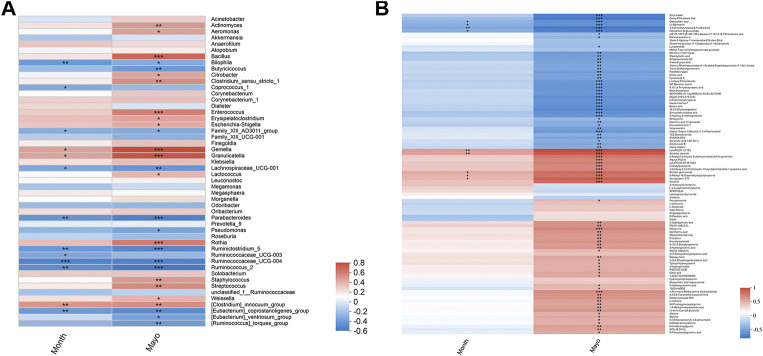
Spearman correlation analysis of candidate genera and metabolites with UC clinical indexes. **(A)** Spearman correlation of candidate genera with UC Mayo score and the course of disease (month). **(B)** Spearman correlation of candidate metabolites with UC Mayo score and the course of disease (month). Different colors represent the value of correlation coefficient, red indicates positive correlation and blue indicated negative correlation. *0.01 < *p* ≤ 0.05，**0.001 < *p* ≤ 0.01，***p ≤ 0.001.

To explore whether mesalamine restored gut microbiota and metabolites were related to UC clinical features, Spearman correlation analysis was performed. As shown in Figure 5A total of 26 genera (such as *Bacillus, Butyricicoccus* and *Streptococcus*) was significantly correlated with both Mayo score and the course of disease (month). Interestingly, the genera decreased by mesalamine in UC patients were positively correlated with Mayo score and the course of disease, and vice versa. For instance, the relative abundance of *Bacillus, Enterococcus* and *Streptococcus* reduced by mesalamine exhibited a significant positive correlation with Mayo score and the course of disease. Whereas, *Butyricicoccus, Parabacteroides* and *Pseudomonas* were increased by mesalamine and had a negative correlation with Mayo score and the course of disease. The results indicated mesalamine might exert a beneficial role in UC by restoring the gut microbiota perturbance.

Besides, most of the candidate metabolites (85/102) revealed a significant correlation with UC Mayo score (Figure 5B). It was worth noting that the metabolites decreased by mesalamine were also positively correlated with Mayo score, and vice versa. For example, the levels of ophthalmic acid, allysine and styrene were reduced by mesalamine and positively correlated with Mayo score. Whereas, several metabolites increased by mesalamine such as cholic acid, deoxycholic acid and enoxolone exhibited negative correlation with Mayo score. Interestingly, the correlation pattern of metabolites with Mayo score was consistent with that of gut microbiota, which suggested that the perturbance of gut microbiota might correlate with metabolites disturbance in UC.

### Gut Microbiota Correlated with Metabolites Changes in Different Pathways

**FIGURE 6:**
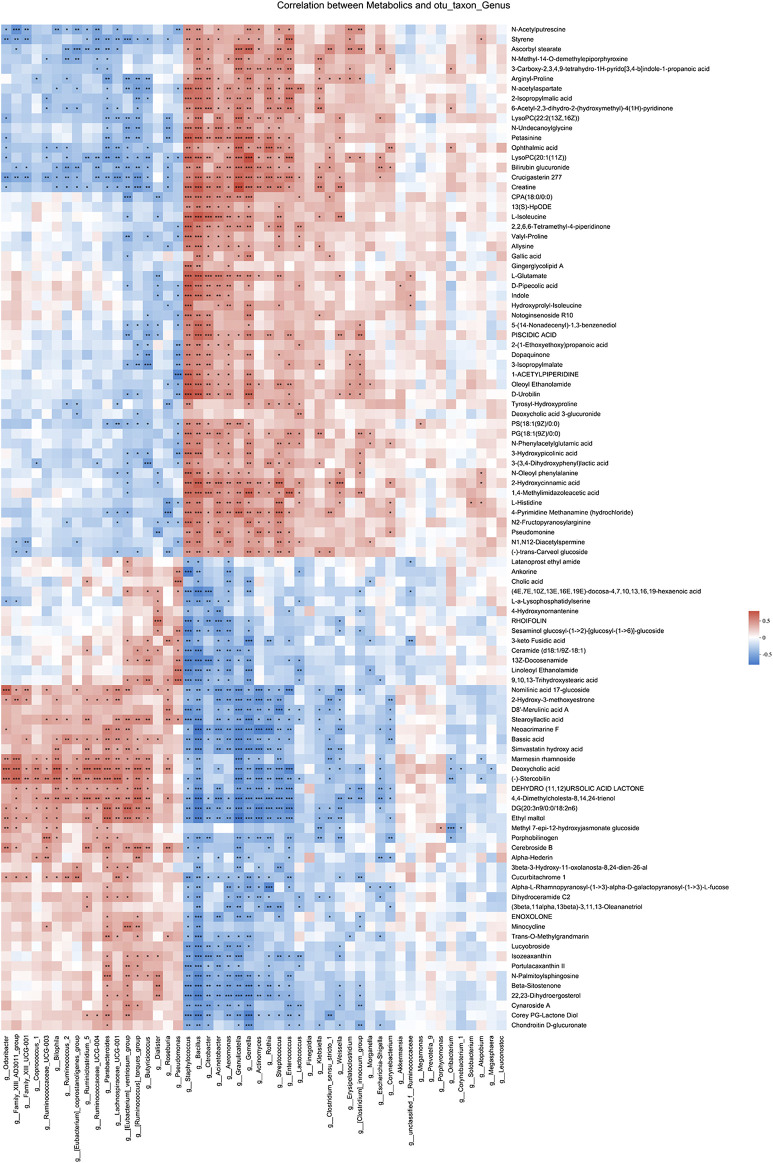
Spearman correlation between candidate genera and metabolites. Different colors represent the value of correlation coefficient, red indicates positive correlation and blue indicated negative correlation. *0.01 < *p* ≤ 0.05，**0.001 < *p* ≤ 0.01，***p ≤ 0.001.

Spearman correlation analysis was also performed for 49 genera and 102 metabolites candidates to examine whether there is a correlation of gut microbiota with metabolites changes (Figure 6). Of interest, a batch of mesalamine increased metabolites were negatively correlated with mesalamine decreased genera, and vice versa. The metabolites such as ophthalmic acid and styrene that were reduced by mesalamine were positively correlated with mesalamine decreased *Enterococcus* genus, and mesalamine increased metabolites such as deoxycholic acid and enoxolone were positively correlated with mesalamine increased genera *Butyricicoccus* and *Parabacteroides*, respectively.

**FIGURE 7:**
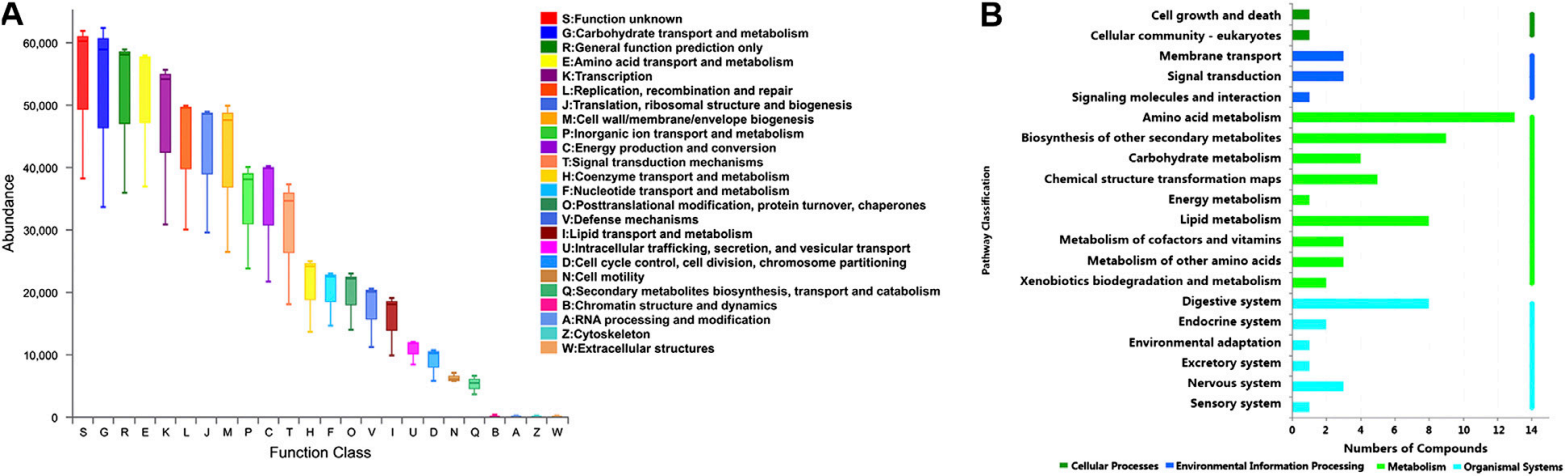
Pathway analysis of candidate genera and metabolites. **(A)** The candidate genera functional prediction by PICRUSt analysis. The horizontal axis represents the function number, and the vertical axis represents the functional abundance. **(B)** Pathway enrichment of candidate metabolites. The horizontal axis represents the number of enriched metabolites, and the vertical axis represents the enriched pathway items.

Furthermore, the functional correlation between gut microbiota and metabolites changes was explored. Using PICRUSt analysis, the 49 candidate genera were enriched in 24 function classes including carbohydrate transport and metabolism, amino acid transport and metabolism, lipid transport and metabolism, signal transduction mechanisms, secondary metabolites biosynthesis, transport and catabolism, etc (Figure 7A). The 102 candidate metabolites were enriched in 14 KEGG pathway related items such as amino acid metabolism, biosynthesis of other secondary metabolites, lipid metabolism, carbohydrate metabolism, etc (Figure 7B). We observed that the candidate genera and metabolites were enriched in many similar molecular pathways such as amino acid metabolism and secondary metabolites biosynthesis, which might implicate the high functional correlation of gut microbiota with related metabolites.

### The Gut Microbiota Signature Discriminate Treatment-Naïve UC Patients from Both Normal Healthy Control and Mesalamine-Responded UC Patients

**FIGURE 8:**
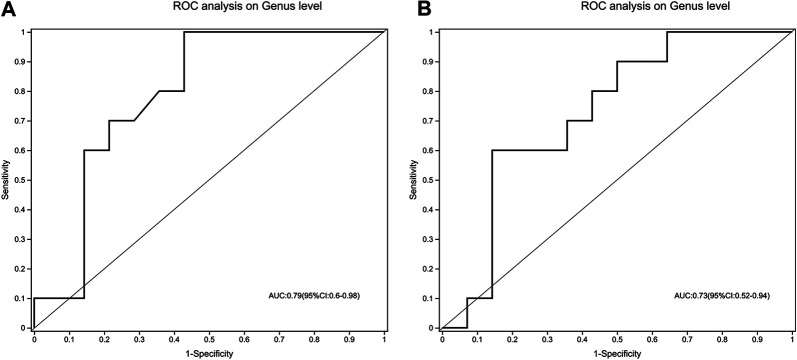
A signature composed of five genera discriminating UC group from both NC and mesalamine groups. **(A)** ROC curve analysis of UC group and NC group. **(B)** ROC analysis of UC group and mesalamine group.

To evaluate whether gut microbiota could be used to distinguish treatment-naïve UC patients from normal healthy control or mesalamine-responded UC patients, ROC analysis was performed for 49 candidate genera. Our results indicated that a gut microbiota signature composed of five genera including *Escherichia-Shigella, Streptococcus, Megamonas, Prevotella_9* and [*Eubacterium*]*_coprostanoligenes _group* might be used to distinguish treatment-naïve UC patients from normal healthy controls (AUC = 0.79, 95% CI 0.6–0.98, Figure 8A). Meanwhile, the five genera signature might also be used to discriminate treatment-naïve UC patients from mesalamine-responded UC patients (AUC = 0.73, 95% CI 0.52–0.94, Figure 8B).

## Discussion

In the present study, 16S rRNA sequencing and LC-MS metabolomics were integrated to detect the perturbance of gut microbiota and metabolites in UC patients and observed the effect of mesalamine. We observed there were significant changes of gut microbiota and metabolites in UC patients, and mesalamine might exert beneficial effects in UC by partially restoring gut microbiota and correlated metabolites in different pathways.

The important role of gut microbiota in UC has been widely accepted, and disruptions to the microbiome have been implicated in the pathogenesis (Derikx et al., 2016). A recent study of fecal microbiota analysis revealed that the relative abundance of *Escherichia-shigella* and *Streptococcus* was elevated, whereas *Bacteroides* and *Prevotella_9* was reduced in UC patients compared to healthy participants (Sun et al., 2019). Another study reported the gut microbial dysbiosis in Chinese inflammatory bowel disease patients, and found that the community of Megamonas and *Butyricicoccus*, which could produce short-chain fatty acids and modulate colonic regulatory T cells, was significantly repressed in UC patients stool samples (Ma et al., 2018). Here, we also observed significant changes of gut microbiota composition in treatment-naïve UC patients using 16S rRNA sequencing bacteria community analysis. We noted that the relative abundance of many genera such as *Escherichia-shigella, Megamonas, Clostridium_sensu_stricto_1, Enterococcus* and *Citrobacter* was increased*,* while a batch of genera such as *Megamonas, Prevotella_9, Parabacteroides [Eubacterium]_ventriosum_ group, Ruminococcus_2, Roseburia, Butyricicoccus, Dialister, Akkermansia* and [*Eubacterium*]*_coprostanoligenes_group* was reduced in UC patients. The results were partially consistent with previous studies. Importantly, we observed that the relative abundance of 49 candidate genera was significantly reversed by mesalamine intervention. It is interesting that most of the candidate genera were significantly correlated with Mayo score and the course of disease. Of which, several genera such as *Enterococcus* and *Streptococcus* exhibited a significant positive correlation with Mayo score and the course of disease, exerting their adverse effect in UC pathogenesis. Whereas, other genera such as *Butyricicoccus, Parabacteroides* and *Pseudomonas* were negatively correlated with Mayo score and the course of disease, which implicated their beneficial role in UC. It has been reported the amount of *Enterococcus* was higher in UC patients than in healthy subjects (Nemoto et al., 2012). Another longitudinal analyses of gut mucosal microbiotas in UC patients revealed that high clinical activity indices and sigmoidoscopy scores were associated with *Enterococcus faecalis* (Fite et al., 2013). Our results were partially consistent with these data. However, there were conflicting results concerning the role of *Streptococcus* in UC*.* For example, it is suggested that infection with highly-virulent specific types of *Streptococcus mutans* might be a potential risk factor in the aggravation of UC (Kojima et al., 2012), whereas, it has been reported that *Streptococcus thermophilus* strain might have the potential to reduce signs of colitis (Bailey et al., 2017). These results indicated that specific strain of *Streptococcus* might have different role in the development of UC. In the present study, we reported the association of *Streptococcus* genus level with UC and did not examine the specific *Streptococcus* species. Further extensive investigation of the identified candidate genera may obtain novel gut microbiota targets for UC treatment.

It is noted that the perturbation of gut microbial community may lead to metabolite alterations. The disordered metabolites may enter the host, then modulate intestine epithelial cells and inflammation in the disease progression (Arpaia et al., 2013). Therefore, we examined the metabolite profiles across fecal samples from normal healthy subjects, treatment-naïve UC patients and mesalamine-responded UC patients. By LC-MS metabolomics, we noted that a batch of metabolites were significantly different in treatment-naïve UC patients, and mesalamine restored the changes of 102 candidate metabolites. Many amino acids in serum such as leucine, isoleucine, glycine and histidine have been implicated in inflammatory bowel diseases (Dawiskiba et al., 2014; Probert et al., 2018). In the present study, l-isoleucine and l-histidine in fecal samples were remarkably changed in treatment-naïve UC patients and could be restored by mesalamine intervention. The findings indicated their potential role in UC and might serve as potential therapeutic targets. Dysmetabolism of bile acids has also been found in UC pathogenesis (Pavlidis et al., 2015). Duboc, et al., 2013 reported that the level of secondary bile acids was decreased in fecal and serum samples of UC patients, and Sinha, et al., 2020 observed lithocholic acid and deoxycholic acid were reduced in stool samples of UC patients (Duboc et al., 2013; Sinha et al., 2020). Our results partially conformed to their findings. We noted that cholic acid and deoxycholic acid concentrations were reduced in UC patients and could be restored by mesalamine intervention. Besides, we also obtained many other mesalamine reversed candidates which had been implicated in UC. For example, styrene is a benzenoid compound produced by decarboxylation of cinnamic acid and has been reported positively correlated with disease activity in UC (De Preter et al., 2015). In present study, we noted that the level of styrene was higher in treatment-naïve UC patients than normal healthy controls and positively correlated with UC Mayo score. It is worth noting that most of the candidate metabolites were not only significantly correlated with UC Mayo score, but also correlated with specific bacteria genera. Furthermore, we observed that the candidate genera and metabolites were enriched in many similar KEGG pathways such as amino acid metabolism and secondary metabolites biosynthesis, indicating their high functional correlation. The results suggested that mesalamine might exert a beneficial role in UC by modulating gut microbiota genera and relevant metabolites in different pathways. In-depth extensive investigation of the identified candidates may provide us novel targets for UC treatment.

Finally, we identified a gut microbiota signature composed of five genera that might be used to discriminate treatment-naïve UC patients from both normal healthy controls and mesalamine-responded UC patients. However, our study has some inherent limitations. Firstly, the findings in the present study were based on the limited samples with no further functional experiments, and the identified genera and metabolites candidates should be further verified in a comprehensive and large scale investigation. Second, this exploratory study composed of two cross-sectional trials. Current results could only support correlation, not the causality. Besides, the cross-sectional design ignored the pre-treatment clinical information of mesalamine group. Potential imbalance may exist between UC group and mesalamine group, which may affect the results. Longitudinal design should be considered in future studies. Third, the course of disease might affect the gut microbiota and metabolites, and further study should be performed in UC patients with matched course of disease to validate our findings.

## Conclusion

In summary, 16S rRNA sequencing and metabolomics approaches were integrated to detect the perturbance of gut microbiota and metabolites in stool samples across normal healthy controls, treatment-naïve UC patients and mesalamine-responded UC patients. We observed significant changes of gut microbiota and metabolites in UC patients. Mesalamine might exert beneficial effects in UC by modulating gut microbiota and correlated metabolites in different pathways. We also identified a gut microbiota signature to discriminate treatment-naïve UC from normal healthy controls and mesalamine-responded UC patients. Our results may shed new lights on the mechanism of mesalamine in UC treatment and provide us novel therapeutic targets.

## Data Availability

The datasets presented in this study can be found in online repositories. The names of the repository/repositories and accession number(s) can be found below: NCBI SRA database, under the accession (BioProject PRJNA681685).
